# Supplementary material for: Distributed subthreshold representation of sharp wave-ripples by hilar mossy cells
Source: eLife. 2025 Oct 7;14:e97270. doi: 10.7554/eLife.97270 (PMC12503485; doi:10.7554/eLife.97270)
Supplement: Supplementary file 1. — Some data were recorded multiple times from a single slice, and the quality of the recordings was confirmed by checking the access resistance and intrinsic electrophysiological properties of the mossy cells (MCs) did not change significantly between recordings. When there were multiple recordings, each value was entered in the same cell. The data thus obtained were concatenated in machine learning and treated as a single dataset. Note that 190801_2 and 190927_1 have overlapping slice IDs and different cell numbers. In this case, the MCs to be recorded are common, but the recording times (i.e. SWR events) used for the actual analysis do not overlap. [file elife-97270-supp1.docx]

**Supplementary File 1. Recording time and number of SWR events in each slice**

| Mouse ID | Slice ID | Recorded MCs | Recording time (s) | Number of SWR | SWR frequency (Hz) |
| --- | --- | --- | --- | --- | --- |
| 1 | 190702_1 | 3 | 180 / 307.2 | 81 / 182 | 0.45 / 0.59 |
| 2 | 190719_2 | 4 | 303.67 | 192 | 0.63 |
| 3 | 190725_1 | 3 | 308.58 / 311.65 | 150 / 169 | 0.49 / 0.54 |
| 4 | 190725_2 | 4 | 301.88 | 185 | 0.61 |
| 5 | 190726 | 5 | 300.85 | 180 | 0.6 |
| 6 | 190731_1 | 3 | 306.58 / 314.93 | 144 / 153 | 0.47 / 0.49 |
|  | 190731_2 | 3 | 301.21 | 198 | 0.66 |
|  | 190731_3 | 4 | 301.67 / 329.06 / 140 | 262 / 265 / 123 | 0.87 / 0.81 / 0.88 |
| 7 | 190801_1 | 3 | 319.69 | 236 | 0.74 |
|  | 190801_2 | 4 | 301.52 | 143 | 0.47 |
|  | 190801_2 | 5 | 256 | 93 | 0.36 |
|  | 190801_3 | 3 | 301.41 | 272 | 0.90 |
| 8 | 190823_1 | 3 | 300.75 / 170 | 231 / 239 | 0.77 / 1.41 |
| 9 | 190823_3 | 4 | 301 / 301 / 290 | 320 / 310 / 315 | 1.06 / 1.03 / 1.08 |
| 10 | 190902 | 3 | 314.11 / 322.25 | 154 / 186 | 0.49 / 0.58 |
| 11 | 190905 | 3 | 285 | 128 | 0.45 |
| 12 | 190910 | 3 | 211.72 / 124 | 118 / 65 | 0.56 / 0.52 |
| 13 | 190924_1 | 4 | 301.8 / 300.85 / 300.6 | 176 / 151 / 154 | 0.58 / 0.5 / 0.51 |
|  | 190924_2 | 3 | 301.1 / 300.65 | 272 / 277 | 0.89 / 0.92 |
|  | 190924_3 | 4 | 56 / 306.12 | 28 / 235 | 0.5 / 0.77 |
|  | 190924_4 | 3 | 301.06 / 301 | 268 / 200 | 0.89 / 0.66 |
| 14 | 190927_1 | 3 | 190.34 / 357 | 194 / 271 | 1.02 / 0.76 |
|  | 190927_1 | 4 | 120 / 110 | 98 / 95 | 0.82 / 0.86 |
|  | 190927_2 | 3 | 301.62 / 301.41 | 211 / 317 | 0.7 / 1.05 |
|  | 190927_3 | 3 | 160 / 301 / 300.49 | 47 / 146 / 55 | 0.29 / 0.48 / 0.18 |

Some data were recorded multiple times from a single slice, and the quality of the recordings was confirmed by checking the access resistance and intrinsic electrophysiological properties of the MCs did not change significantly between recordings. When there were multiple recordings, each value was entered in the same cell. The data thus obtained were concatenated in machine learning and treated as a single data set.

Note that 190801_2 and 190927_1 have overlapping slice IDs and different cell numbers. In this case, the MCs to be recorded are common, but the recording times (i.e. SWR events) used for the actual analysis do not overlap.
